# Effect of risperidone on morphine‐induced conditioned place preference and dopamine receptor D2 gene expression in male rat hippocampus

**DOI:** 10.1002/brb3.2975

**Published:** 2023-04-11

**Authors:** Zahra Mansouri Arani, Nasrin Heidariyeh, Gholamreza Ghavipanjeh, Majid Lotfinia, Hamid Reza Banafshe

**Affiliations:** ^1^ Physiology Animal, Department of Biology, Faculty of Sciences, Qom Branch Islamic Azad University Qom Iran; ^2^ Department of Biology, Faculty of Sciences, Qom Branch Islamic Azad University Qom Iran; ^3^ Department of Physiology Kashan University of Medical Sciences Kashan Iran; ^4^ Department of Biotechnology, Physiology Research Center, Basic Sciences Research Institute Kashan University of Medical Sciences Kashan Iran; ^5^ Department of Addiction Studies, School of Medicine Kashan University of Medical Sciences Kashan Iran

**Keywords:** conditioning, dopamine D2 receptor, hippocampus, morphine dependence, real‐time PCR, risperidone

## Abstract

**Background:**

Previous studies suggest the possible effect of risperidone on brain reward system and D1 and D2 dopamine receptors’ involvement in morphine‐induced conditioned place preference (CPP).

**Aims:**

The present study was designed to investigate the effect of risperidone as an atypical antipsychotic drug on morphine‐induced CPP and D2‐like dopamine receptor gene expression in rat.

**Materials and Methods:**

An unbiased CPP paradigm was used to study the effect of risperidone. Intraperitoneal (i.p.) injection of risperidone (1, 2, and 4 mg/kg) was performed 30 min before the morphine (10 mg/kg, i.p.) injection and just after the rat was placed in the CPP box. The open field test was used to assay the locomotor activity of animal. The gene expression of D2 dopamine receptor in hippocampus was measured by real‐time PCR technique. The hippocampi of rats were also used for histology evaluation.

**Results:**

Morphine‐produced (10 mg/kg) CPP and morphine‐induced CPP were reversed only by the administration of a low dose of risperidone (1 mg/kg). Low dose of risperidone (1 mg/kg) showed no effect on locomotor activity but a higher dose of risperidone (2 and 4 mg/kg) decreased locomotor activity. Real‐time PCR data analysis revealed that the gene expression of D2 dopamine receptor had significant difference between morphine and a 1 mg/kg dose of risperidone. Moreover, in histological evaluation, apoptosis was observed in the morphine group, whereas there was no evidence of apoptosis in the risperidone‐treated groups.

**Conclusion:**

Our results suggest that risperidone (1 mg/kg) reverses the morphine‐induced CPP and may reduce the rewarding properties of morphine. It is also demonstrated that risperidone decreases the expression of D2 receptor in rat hippocampus. Therefore, risperidone can be considered potential adjunct therapy in morphine dependence.

## Graphical abstract

The effect of risperidone on morphine‐induced CPP and D2‐like dopamine receptor gene expression in rats.

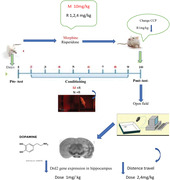

## 1 INTRODUCTION

Drug addiction is a growing problem in the global community that leads to serious physical, psychological, and social complications. Common methods for addiction treatment were not successful, and unfortunately, relapse rates for individuals who enter recovery from a substance dependence are quite high (Moeini et al., 2019). Morphine is the main alkaloid of opium, which has high addictive properties. It is a potent analgesic drug, though its use is limited due to dependence, tolerance, and the risk of abuse (Rothwell et al., 2009). Chronic morphine abuse leads to physical and psychological dependence. Physical dependence on morphine is characterized by withdrawal syndrome that can develop after sudden cessation of drug use (Listos et al., 2019). Morphine withdrawal symptoms in people include craving, anxiety, irritability, perspiration, dysphoria, lacrimation, runny nose, abdominal pain, and diarrhea, and in animals include jumping, paw tremors, teeth chattering, wet dog shakes, and diarrhea (Narita et al., 2001). The most common treatment for morphine dependence is long‐acting opioid agonists such as methadone. Methadone can be very helpful in treating opioid dependence, but it can also be addictive and have a number of side effects, including withdrawal syndrome, cravings, and cognitive impairment (Javdan et al., 2019). Opioids bind to specific opioid receptors in the central and peripheral nervous system and other tissues. There are three major classes of opioid receptors, mu, kappa, and delta (Jia et al., 2011). Morphine causes decreasing neurogenesis in a granular cellular layer of hippocampus in matured rats through undesired dendritic ductility and atrophy or neuronal destruction in hippocampus and associated structures to limbic cortex (Grilli, 2017). As a result of extensive research, the mechanisms and factors associated with opioid dependence have been gradually identified. One of the most important pathways related to opioid dependency is mesocorticolimbic pathway, which is a dopaminergic pathway. The mesocorticolimbic system, extending from the ventral tegmental area to the nucleus accumbens and prefrontal cortex (PFC), comprises a dopamine projection implicated in reinforcement learning. The mesocorticolimbic system is the target of opioids and opioid‐evoked neuroplasticity changes, a cellular mechanism that may underlie the maladaptive behaviors that occur after chronic opioid use (Hyman & Malenka, 2001). The conditioned place preference (CPP) method is a common animal model used to study the rewarding and aversive effects of drugs. Although a number of different designs and apparatuses are used, the basic characteristics of this model include the association of a particular environment with drug treatment (Zarrindast et al., 2003). Systemic administration of morphine results in CPP inducing in rats (Olmstead & Franklin, 1997).

Risperidone, under the brand name Risperdal, belongs to atypical antipsychotic drugs group, which can be used as tablet, syrup, and injection. It has been shown to improve both positive and negative symptoms in the treatment of schizophrenia (Horacek et al., 2006). Risperidone is the postsynaptic blocker of dopamine and serotonin receptors. It blocks several receptors in central nervous system, including dopamine type II, serotonin type II, and androgen alpha‐2 adrenergic receptors (Seeman, 2006). The most important adverse effect of risperidone is weight gain, which is more pronounced in youths than in adults. Other common side effects of risperidone include extrapyramidal symptoms, prolactin elevation, and sedation. Risperidone can increase the corrected QT (QTc) interval, although clinically relevant QTc prolongation is rare (Kloosterboer et al., 2021). The present study was designed to evaluate the effect of risperidone on morphine‐induced CPP and dopamine D2 receptor gene expression in the hippocampus of male rats.

## 2 METHODS AND MATERIALS

### 2.1 Animal

The experiments were performed on male Sprague–Dawley rats (230–280 g). They were housed four per cage, in a room under controlled temperature (25 ± 1°C), humidity (38% ± 2%), and lighting (12/12 h light/dark cycle), with food and water available ad libitum. All experiments were approved by the ethical committee of Qom Azad University of Medical Sciences (code: IR.IAU.QOM.REC.1400.078) and followed the European Commission Directive (86/609/EEC) for animal experiments. In order to more adaption to laboratory environment, the animals were placed in the above condition 1 week before the start of the study.

### 2.2 Drugs

Morphine sulfate (Sigma‐Aldrich) was provided by the deputy of food and drug administration. It was used for intraperitoneal injection with the effective dose of 10 mg/kg, which is recommended by different studies as the best effective dose for mental dependence on morphine through the CPP method (McKendrick et al., 2020). Risperidone was supplied by Merck Company as powder. In order to prepare the solvent, 20 mg of risperidone was combined with 2.5–3 cm^3^ DMSO. Then 5 cm^3^ of distilled water and a few drops of Tween 20 were added to become a suspension for intraperitoneal injection.

### 2.3 Study design

Morphine was injected intraperitoneally (i.p.) with the effective dose of 10 mg/kg. Risperidone was administrated as suspension with the dose of 1, 2, and 4 mg/kg (i.p.).

In order to control the effect of risperidone solvent on morphine‐induced CPP, risperidone solvent was injected during CPP on days 2, 4, 6, 8, and 9 and 30 min before the effective dose of morphine administration (control groups). Then they were placed into the apparatus. No administration was done on days 1 and 10. In risperidone treatment group, risperidone was administered with the dose of 1, 2, and 4 mg/kg in each group during CPP on days 2, 4, 6, 8, and 9 and 30 min before the effective dose of morphine. They were placed into the CPP apparatus, and no administration was done on days 1 and 10. Motor activity test in open field apparatus was done 10 min after the CPP test, for each rat in each group.

### 2.4 Place preference conditioning procedure

In all experiments, the CPP procedure was used to examine the effects of risperidone on morphine‐induced place preference. All place conditioning procedures used a biased, counterbalanced conditioning protocol as described previously (Ahmadian et al., 2019; Listos et al., 2016).

#### 2.4.1 Apparatus

The condition place preference apparatus used for place preference induction contains three chambers (A–C). A and B chambers that are related to chamber C by guillotine doors have the same size with the dimension of 30 × 30 × 40 cm^3^. The walls of chamber A are decorated with black and white longitudinal stripes, and its floor is flat, whereas the walls of the chamber B are decorated by black and white transverse stripes to have environmental impacts on rats with morphine injection. The C chamber has the dimension of 40 × 15 × 30 cm^3^ and black walls, which is located between the other two chambers. A camera connecting to monitor is on the top of the apparatus. It records the time when the rat enters each chamber, and finally, it calculates the total time spent in each chamber and the numbers of entrances used for control of rat's behavior. The apparatus was placed in a room lit by a 60‐watt bulb. CPP procedure can be done in two ways: biased and unbiased. We use the biased method in our research. In this method, the animal, according to its interest, becomes conditioned (addicted) to the house in which it is less interested. But in the unbiased method, this point is not considered, and the animals are divided into two equal groups, and each animal becomes conditioned to a separated part of A or B. The CPP protocol lasted 10 days (Ahmadian et al., 2019) and included three stages: preconditioning, conditioning, and post‐conditioning.

#### 2.4.2 Preconditioning phase

On the first day, the doors between the chambers were raised, and the rat was freely allowed to enter A, B, and C chambers for 15 min. The time spent in each chamber was recorded in order to determine the rat's tendency to each chamber called place preference.

#### 2.4.3 Conditioning phase

Conditioning stage occurred on days 2–9 and consisted of eight sessions of 30 min. Morphine and saline were injected alternatively, in morning for every animal, so that rats given morphine in the morning were given saline in the opposite chamber, and contrariwise on subsequent days. For control group that had to receive risperidone solvent and treatment group with risperidone, the intraperitoneal injection of risperidone or its solvent was performed 30 min before saline or morphine administration, and then, the rats were placed in related places.

#### 2.4.4 Post‐conditioning or testing phase

In the post‐conditioning trial (day 10), a test for CPP was given. Animals were placed in the C chamber while the guillotine doors were removed, and like day 1, they were allowed free access to the entire apparatus for 15 min. In this phase, also the time spent in each chamber was recorded (Hosseini et al., 2011). The change in rat preference was calculated as the difference (in seconds) between the times spent in morphine receiving chamber on days 10 and 1 (You et al., 1998).

#### 2.4.5 Open‐field apparatus

This apparatus consists of a rectangle with the dimension of 45 × 45 × 35 cm^3^ made of black metal, and the floor is divided into 25 squares by the lines. The animal movement with all four limbs from one part to another one was considered a unit of movement. The rat's movements were recorded by a camera located at the top of the apparatus and connected to the computer. First, each rat was placed at the center of the apparatus, and its activity was recorded for 5 min. Then some behavioral parameters, including the total distance covered, duration of movement, lack of movement, and the frequency of standing on two feet, were recorded and analyzed (Méndez et al., 2011).

### 2.5 Histological studies

After the behavioral study, rats’ heads were separated by guillotine under deep anesthesia with chloroform. The right and left hippocampi of 4 of each group were separated and kept at −80°C for molecular and histological studies. Histological studies were performed to observe changes in nerve cells of hippocampus CA1 area. Samples were fixed by formalin 10%, and then, they were dehydrated and soaked in paraffin. In order to histological investigation, tissue sections were cut to a thickness of 5 microns. Hippocampus sections were stained by hematoxylin and eosin and used for histological studies.

### 2.6 Dopamine D2 receptor gene expression

The extraction of RNA from tissue samples was conducted by TRIZOL protocol (Méndez et al., 2011). RNA density was examined at a wavelength of 260 nm, and purity was estimated by absorbance ratio at 260/280. cDNA synthesis was performed using a Labcycler thermocycler manufactured by SensoQuest. cDNA quality assays were measured by horizontal electrophoresis on 1% agarose gel.

The ABI StepOnePlus Real‐Time PCR Sequence Detection System (Applied Biosystems) is according to the default thermocycler program for all genes. Real‐time PCR reactions were carried out using replication reaction components, including 10 μL master mix, 1 μL primers, 2 μL synthesized cDNA, and 6 μL distilled water. Thermal program of *Drd2* and *GAPDH* genes is as follows: 1 cycle at 95°C for 2 min for initial denaturation, 40 cycles at 95°C for 5 s, and then, at 61°C for 25 s. The sequence and characteristics of dopamine receptor D2 (*Drd2*) and *GAPDH* genes are listed in Table 1.

**TABLE 1:** The sequences of specific primers for real‐time PCR

Annealing (°C)	Amplicon size (bp)	Sequence	Gene accession number	Primer
61	241	5′CCTGCACCACCAACTGCTTAG‐3′	051339.1	*GAPDH*
		5′GCCAGTGAGCTTCCCGTTCAG‐3′		
61	94	5′‐AGACACCACTCAAGGGCAAC‐3′	051343.1	*Drd2*
		5′‐CGCCTGTTCACTGGGAAACT‐3′		

### 2.7 Analyzing statistical data

Data statistical analyses were done using Prism software. To analyze and compare the groups, the one‐way variance (ANOVA) and Tukey test were used. Data were displayed based on the mean ± standard error (mean ± SEM), and *p* < .05 was considered significant.

## 3 RESULTS

### 3.1 Conditioned place preference induction by morphine

**FIGURE 1:**
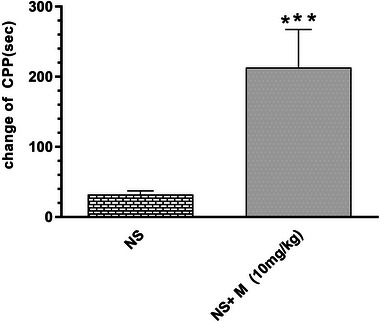
The effect of 10 mg/kg morphine on conditioned place preference (CPP) in male rats. Morphine (10 mg/kg) showed significant difference with normal saline (*p* < .001). The results are based on mean ± SEM, *N* = 8, ***p < .001.

In the first step, the rats were divided into saline and morphine (10 mg/kg) groups for the CPP test. Data analyses showed that receiving the dose of 10 mg/kg morphine leads to more spending time in morphine part than saline part, and CPP was induced significantly (*p* < .001) (Figure 1).

### 3.2 The effect of risperidone on morphine‐induced CPP

**FIGURE 2:**
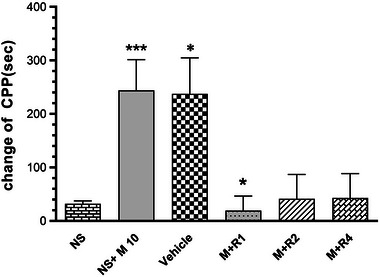
The effect of different dose of risperidone on conditioned place preference (CPP) in male rats. The results present as mean ± SEM, *N* = 8. Morphine (10 mg/kg) showed significant difference with normal saline group (***p < .001). Risperidone (1 mg/kg) decreased CPP in comparison with vehicle group (*p < .05). NS, normal saline; M, morphine (10 mg/kg); R1, risperidone (1 mg/kg); R2, risperidone (2 mg/kg); R4, risperidone (4 mg/kg).

In the second step of study and simultaneously with receiving the effective dose of morphine for the induction of CPP, different doses of risperidone (1, 2, and 4 mg/kg) were injected. A volume of 1 mg/kg dose of risperidone decreased CPP in comparison with morphine and control group (*p* < .05). Higher dose of risperidone (2 and 4 mg/kg) did not show any significant difference between control and morphine groups (Figure 2).

### 3.3 Risperidone effect on locomotor activity

**FIGURE 3:**
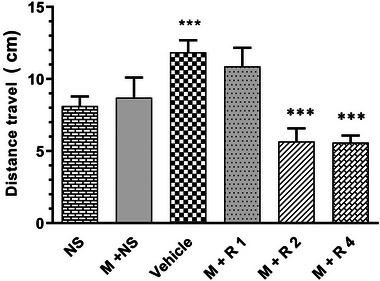
The effect of different dose of risperidone on locomotor activity in male rats. The results presented as mean ± SEM (*N* = 8). Risperidone (2 and 4 mg/kg) had significant difference with control and morphine groups and show lower locomotor activity (***p < .001). NS, normal saline; M: morphine (10 mg/kg); R1, risperidone (1 mg/kg); R2, risperidone (2 mg/kg); R4, risperidone (4 mg/kg).

The results indicated that the groups with higher doses of risperidone (2 and 4 mg/kg) had significant differences between control and morphine groups and show lower locomotor activity (Figure 3). A volume of 1 mg/kg dose of risperidone showed no significant difference between morphine and control group.

### 3.4 Histological studies

Qualitative results of histological studies in morphine and control groups showed more stained nucleus cells and cellular apoptosis whereas these impacts were not observed in treatment with risperidone group.

#### 3.4.1 The effects of morphine on hippocampus histology

**FIGURE 4:**
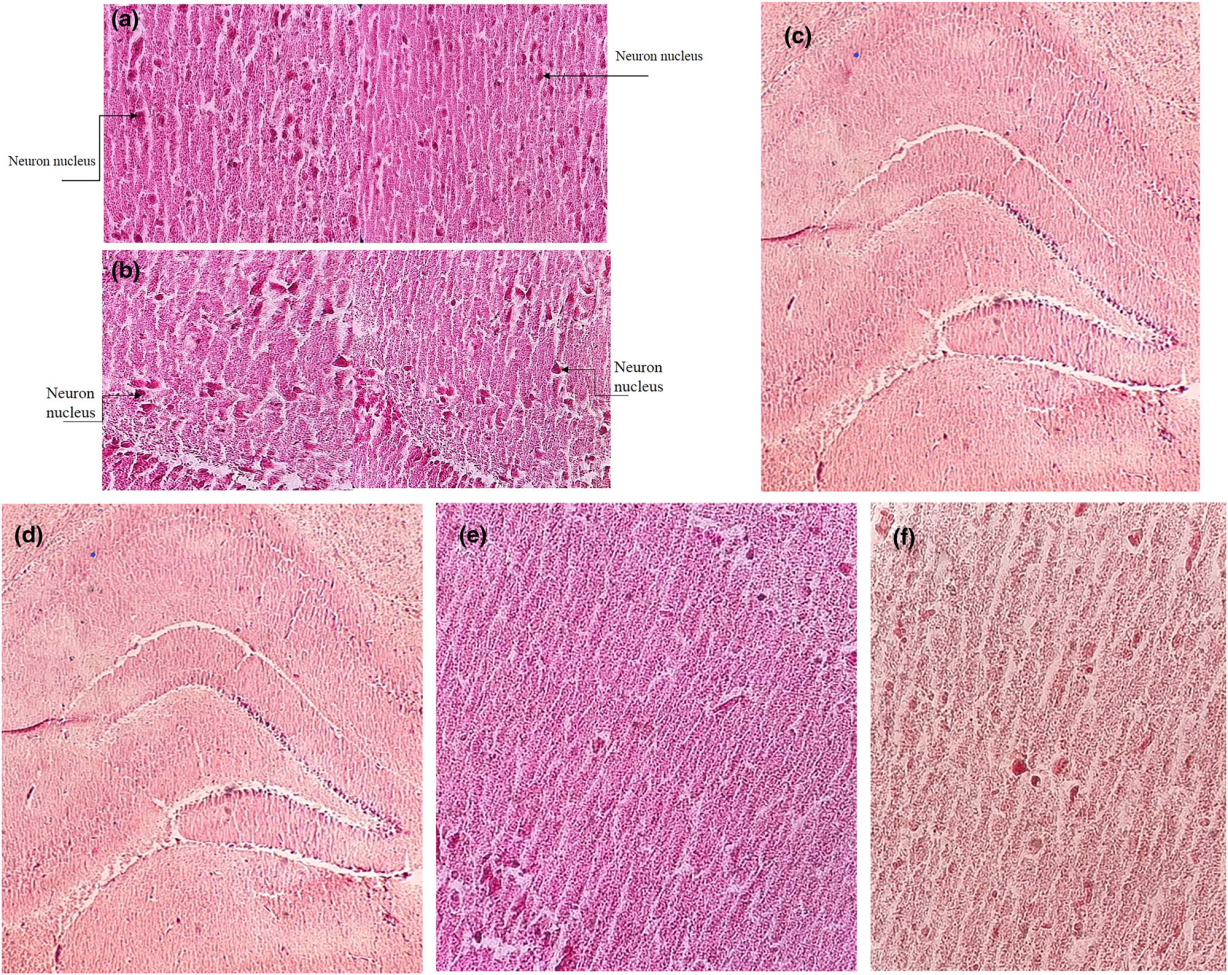
(a and b) The effects of morphine (10 mg/kg) on neurons in CA1 hippocampus area in the morphine group (a) and morphine + risperidone solvent group (b) with the magnification of 400×. (c–f) The effect of risperidone and morphine (10 mg/kg) on neurons in CA1 hippocampus area in the group with 1 mg/kg dose of risperidone (c and d), 2 mg/kg dose of risperidone (e), and 4 mg/kg dose of risperidone (f) with the magnification of 400×.

Histological evaluation of the effects of morphine (10 mg/kg) on the hippocampus shows small, fragmented hyperchromatic neurons in the CA1 region of the hippocampus, indicating cellular apoptosis. As shown in Figure 4, the morphine group had more staining of nuclei and apoptotic cells in comparison with control group (Figure 4a,b).

#### 3.4.2 The effect of risperidone and morphine on hippocampus histology

Histological studies of the CA1 region of hippocampus showed normal cells in risperidone groups (1, 2, and 4 mg/kg). Hippocampal neurons in the morphine group showed cell death, but no cell death was observed in the different risperidone groups (Figure 4c–f). These results indicate the protective effect of risperidone against morphine‐induced apoptosis.

### 3.5 Risperidone effect on *Drd2* gene expression in hippocampus

**FIGURE 5:**
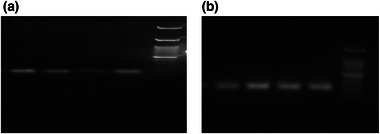
cDNA‐synthesized validation was assessed by primers: (a) (fragment of *Drd2* gene) and (b) (fragment of *GAPDH* gene). Relative expression of *Drd2* gene in hippocampus area of male rats was assessed by real‐time PCR technique.

The expression level of *Drd2* gene considering *GAPDH* as internal control was examined through real‐time PCR. The specific primer for *Drd2* gene was designed, and *GAPDH* primer was picked up from Authentic articles (Table 1). After RNA extraction, cDNA synthesis and observation in agarose gel and the size of desire fragments of *Drd2* and *GAPDH* were confirmed. The size of *GAPDH* and *Drd2* were 241 and 94 base pairs, respectively. Real‐time PCR results proved that the reduplication of *Drd2* and *GAPDH* genes was done perfectly (Figure 5).

**FIGURE 6:**
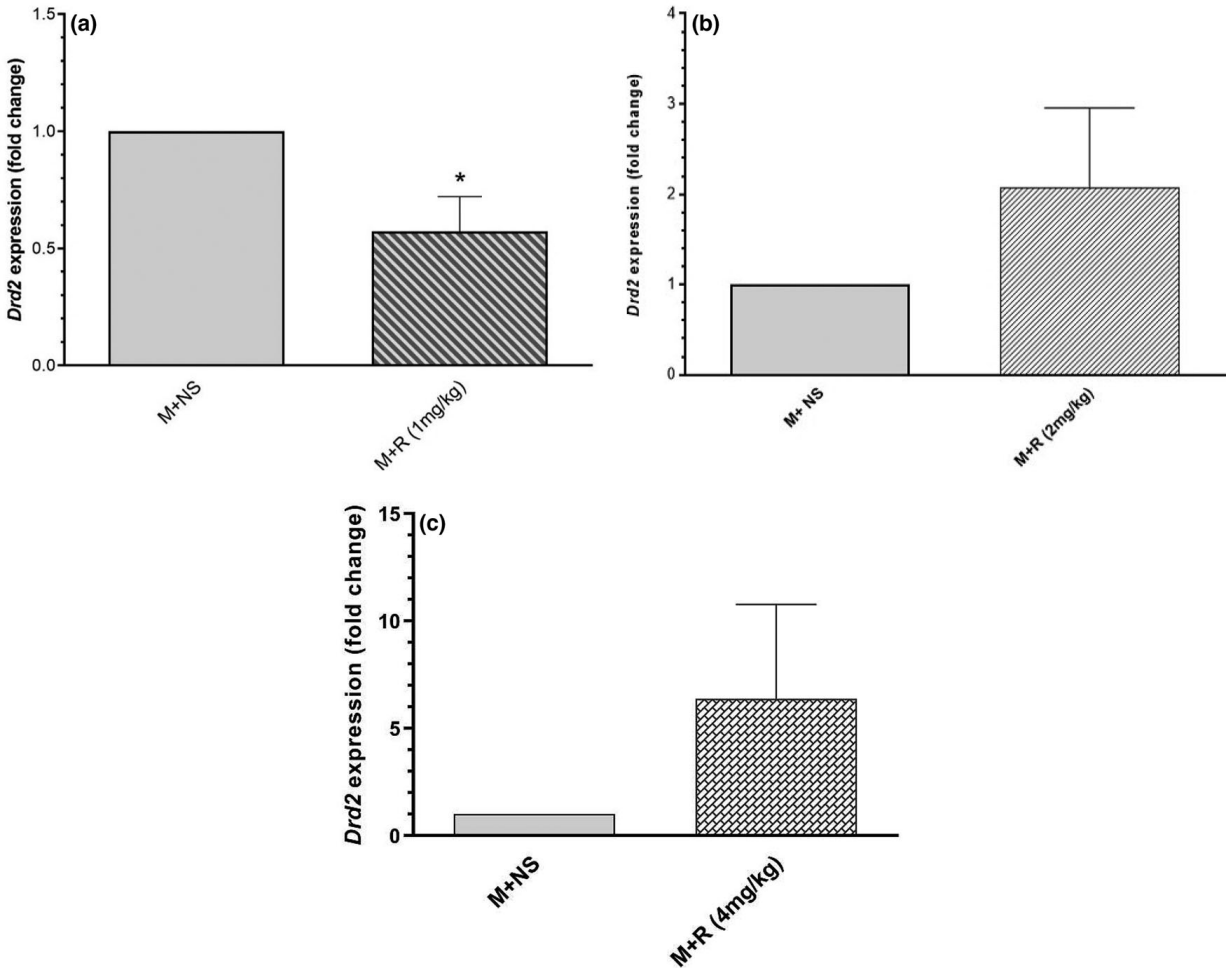
(a) The comparison of *Drd2* gene expression between 1 mg/kg dose of risperidone with effective dose of morphine and control groups. The results are displayed based on the mean ± SEM (*N* = 4, *p < .05) was considered significant. (b) The comparison of *Drd2* gene expression between 2 mg/kg dose of risperidone and effective dose of morphine. Results are displayed based on the mean ± SEM (*N* = 4). No significant difference was seen between two groups (*p* > .05). (c) The results are displayed based on the mean ± SEM (*N* = 4), and *p* < .05 was considered significant. Part (c) showed the comparison of *Drd2* gene expression between 4 mg/kg dose of risperidone and morphine group. Not significant difference was seen between two groups (*p* > .05).

Fold change charts confirmed that a 1 mg/kg dose of risperidone significantly decreased the dopamine receptor D2 gene expression (Figure 6a), but 2 and 4 mg/kg doses of risperidone did not show significant difference in dopamine D2 gene expression compared to morphine group with the effective dose of 10 mg/kg. These results proved that treatment with 1 mg/kg risperidone and morphine at the same time significantly decreased dopamine receptor D2 expression level in hippocampus. However, in treatment with 2 and 4 mg/kg of risperidone and morphine, no significant difference was seen (Figure 6b,c).

## 4 DISCUSSION

Drug addiction is related to functional disorders of many brain systems, including memory and cognitive and motivational systems (Niu et al., 2013). CPP is one of the protocols used for drug reward studies (Niu et al., 2013). Morphine like many other drugs can make CPP (Kargari et al., 2012).

Risperidone is one of the optional medicines for autism treatment (Pandina., Bossie & Youssef., 2007). This drug affects nervous system by changing neurotransmitters. Moreover, it is proved that the density of anterior brain dopamine transporters in rats treated with risperidone is reduced in ventral, posterior, and interior anterior (Bardgett et al., 2020). The current study investigated the risperidone effects on morphine‐induced CPP and the expression of dopamine receptor D2 gene in hippocampus. The results of current study demonstrated that intraperitoneal injection of morphine‐induced (10 mg/kg) CPP and the rats after receiving morphine showed the desire for morphine receiving place compared to saline receiving place. Moreover, it was confirmed that risperidone (1 mg/kg) injection before morphine in CPP days could interfere in reward systems like dopamine and reduced rewarding properties of morphine. Therefore, risperidone can be considered a new potential therapy for morphine dependence. Most brain‐rewarding drugs enhance the effect of dopamine in the reward pathway by different mechanisms (Cousins et al., 2002). Morphine binds to opioid receptors on GABAergic inhibitory interneurons of ventral tegmental area, reduces cyclic adenosine monophosphate, and inhibits them. Inhibition of these inhibitory interneurons is resulted in increasing dopamine release in the nucleus accumbens (Steffensen et al., 2001). It is proposed that morphine induces reward and CPP through dopamine enhancement in nucleus accumbens. Different brain areas, such as ventral tegmental, nucleus accumbens, and hippocampus, are involved in this phenomenon (Kloosterboer et al., 2021). On the other hand, learning is also involved in the CPP mechanism, because the animal has to make connection between reward and the related place. Memory and learning play a crucial role in drug addiction development. Furthermore, morphine has been shown to improve cognition process (Zarrindast et al., 2003). It is necessary to mention that CPP induction by morphine is a complicated phenomenon and different neurotransmitter systems like opioid, dopamine, GABAergic, and serotonergic systems are involved in, and it is based on reward and memory mechanisms that are placed in hippocampus and nucleus accumbens areas (Koob, 1992). These mechanisms are induced in brain during morphine administration and when the animal is placed in the environment where it received morphine before, they are activated and the animal is looking for the drug (Ribeiro Do Couto et al., 2003). Several studies have demonstrated that dopaminergic mesolimbic system plays an important role in morphine‐induced CPP (Moaddab et al., 2009). This system is initiated from the ventral tegmental areas that are actually dopamine neurons and terminated to the nucleus accumbens, forehead cortex, and areas involved in memory like the hippocampus and amygdala. However, Other anatomical pathways and neurotransmitters are involved in dependence induction (Mobasher et al., 2006). Recent studies also proved that among the several dopamine receptors, D2 receptors directly and powerfully increase extracellular dopamine concentration and play an important role in making and expressing morphine‐induced CPP (Rouge‐Pont et al., 2002) so that the inhibition of D2 receptors in neurons can significantly reduce morphine‐induced CPP (Katebi & Haghparast, 2018). Inhibition of morphine effects on reward empowerment and CPP induction by sulpiride prescription (antagonist of dopamine receptor D2) is an important evidence for this assumption (Manzanedo et al., 2001). Due to the ability of opioid drugs for the stimulation of dopamine release in mesocorticolimbic pathway, these drugs can induce long‐term memory of hippocampus. This is the main reason for the memory of drug use in addicted people, which remains for a long time after quitting drug use (Hyman & Malenka, 2001; Mobasher et al., 2006). The results of this study showed that risperidone injection especially in 1 mg/kg dose before morphine injection in CPP induction days can inhibit the induction of CPP by morphine. It was also demonstrated that a 1 mg/kg dose of risperidone reduced the expression of dopamine receptor gene in hippocampus area, which is an evidence of involvement of dopamine D2 receptor. This study also proved that lower doses of risperidone have greater impact on the reduction of brain reward and morphine dependence (Manzanedo et al., 2001). Previous studies have also shown that decreased dopamine activity leads to changes in cognitive function (line et al., 2002). Working memory impairment is also observed after using D2 antagonists. Risperidone is a remarkable antagonist of 5HT2A serotonin and dopamine receptor D2, and its lower doses can alleviate the negative effects of schizophrenia (Goodman & Gilman's, 2014). However, Boman and De Butte (2019) found that risperidone leads to spatial learning reduction. In addition to dopamine, there are different neurotransmitters in hippocampus. Acetylcholine is one of the most important neurotransmitters released from hippocampus, which is revealed from cholinergic cell bodies in medial septal area (Favaroni Mendes & Menescal‐de‐Oliveira, 2008). According to this fact that cholinergic, dopaminergic, and opioid systems are involved in the physical dependence of morphine (Zarrindast & Mousa‐Ahmadi, 1999) and vertical receptors D2 are related to memory function, this function may be performed by the regulation of acetylcholine release (Samuels & Szabadi, 2008). Several studies conducted on animals highlighted that although there is extended and integrated relation between cholinergic and serotonergic systems, they may sometimes have antagonistic functions. Working memory impairment can occur due to the cholinergic system block which is improved through serotonin neurotransmitter empowerment (Marighetto et al., 2008). Risperidone can also inhibit morphine‐induced CPP by affecting cholinergic neurons (Terry et al., 2007).

In the present study, doses of 2 and 4 mg/kg risperidone failed to significantly reduce morphine‐induced CPP, which may be due to the fact that increasing the dose of risperidone alters its effect on dopamine and serotonin receptors. Other studies confirmed that higher doses of risperidone (3 mg/kg) increases 5HT1A receptors in the inner cortex of forehead and hippocampus (Choi et al., 2010). It has also been reported that μ‐opioid receptors can increase serotonin secretion by inhibiting GABAergic neurons in the raphe nucleus (Tao & Auerbach, 2002); on the other hand, high‐dose risperidone may increase dopamine release through serotonergic mechanisms (Harris & Aston‐Jones, 1994). It is also indicated that 2 and 4 mg/kg doses of risperidone decreased the motor activity of rats, which may be due to the drowsiness, the most common side effect of risperidone (Miller et al., 1992). So increasing the dose of risperidone leads to less motor activity, which may be the result of metabolism reduction in hippocampus and forehead cortex (Liddle et al., 2000). Risperidone also causes over the decrease of dopaminergic mesolimbic function in male rats leading to the reduction of movements’ number in motor activity tests (Tendilla‐Beltrán & Meneses‐Prado, 2019).

Histological evaluation of the effects of morphine (10 mg/kg) on the hippocampus shows small, fragmented hyperchromatic neurons in the CA1 region of the hippocampus, indicating cellular apoptosis. However, these results were not seen in groups treated with risperidone. As a result, risperidone can prevent neuronal apoptosis by morphine. Many studies show that amphetamines and heroin induce apoptosis in nerve cells. Earlier studies confirmed that the apoptosis of differentiated neurons can occur because of using these drugs and changing in intercellular dopamine (Jia et al., 2011). Chronic use of morphine induces oxidative stress, inflammation, and apoptosis in brain neurons specially hippocampus neurons of laboratory animals (Shibani et al., 2019). Moreover, using drugs leads to increased dopamine in brain and after several times using in a relatively short period of time results in the destruction of neurons (Takeshi et al., 2011). Risperidone has been proven to improve PFC function by restoring neuroplasticity and reducing oxidative/nitrosative stress (Tendilla‐Beltrán & Meneses‐Prado, 2019), and it is possible that risperidone prevents neuron destruction by reducing dopamine. The current study as well as previous studies confirmed that risperidone decreases dopamine activity by reducing D2 receptors. It is also proved that lower doses of risperidone can block dopamine receptors D2 so that 1 mg/kg dose of risperidone reduces morphine‐induced place preference and also an expression of dopamine D2 receptor gene in hippocampus area and neuron destruction in male rats.

## 5 CONCLUSIONS

Our results showed that risperidone (1 mg/kg) reverses the morphine‐induced CPP and may reduce the rewarding properties of morphine. It is also demonstrated that risperidone decreases the expression of D2 receptor in rat hippocampus. Histological evaluation suggests the protective effect of risperidone against morphine‐induced apoptosis. Therefore, risperidone can be considered potential adjunct therapy for morphine dependence. Given the complex neurobiology of opioid dependence and the involvement of different signaling pathways, further research studies are needed to elucidate the effects of risperidone on morphine dependence. The reduction of morphine‐induced CPP by risperidone is mediated by other mechanisms that should be considered in future studies. In future studies, other pathways such as serotonergic and adrenergic pathways are proposed to investigate. Further studies should also be performed on risperidone doses lower than 1 mg/kg.

## CONFLICT OF INTEREST STATEMENT

The authors declare no conflict of interest.

### PEER REVIEW

The peer review history for this article is available at https://publons.com/publon/10.1002/brb3.2975.

## Data Availability

The data sets used and/or analyzed during the current study are available from the corresponding authors per request.
